# *brca2* and *tp53* Collaborate in Tumorigenesis in Zebrafish

**DOI:** 10.1371/journal.pone.0087177

**Published:** 2014-01-29

**Authors:** Heather R. Shive, Robert R. West, Lisa J. Embree, Champa D. Golden, Dennis D. Hickstein

**Affiliations:** Experimental Transplantation and Immunology Branch, Center for Cancer Research, National Cancer Institute, National Institutes of Health, Bethesda, Maryland, United States of America; UMDNJ-Robert Wood Johnson Medical School, United States of America

## Abstract

Germline mutations in the tumor suppressor genes *BRCA2* and *TP53* significantly influence human cancer risk, and cancers from humans who inherit one mutant allele for *BRCA2* or *TP53* often display loss of the wildtype allele. In addition, *BRCA2*-associated cancers often exhibit mutations in *TP53*. To determine the relationship between germline heterozygous mutation (haploinsufficiency) and somatic loss of heterozygosity (LOH) for *BRCA2* and *TP53* in carcinogenesis, we analyzed zebrafish with heritable mutations in these two genes. Tumor-bearing zebrafish were examined by histology, and normal and neoplastic tissues were collected by laser-capture microdissection for LOH analyses. Zebrafish on a heterozygous *tp53^M214K^* background had a high incidence of malignant tumors. The *brca2^Q658X^* mutation status determined both the incidence of LOH and the malignant tumor phenotype. LOH for *tp53* occurred in the majority of malignant tumors from *brca2* wildtype and heterozygous mutant zebrafish, and most of these were malignant peripheral nerve sheath tumors. Malignant tumors in zebrafish with heterozygous mutations in both *brca2* and *tp53* frequently displayed LOH for both genes. In contrast, LOH for *tp53* was uncommon in malignant tumors from *brca2* homozygotes, and these tumors were primarily undifferentiated sarcomas. Thus, carcinogenesis in zebrafish with combined mutations in *tp53* and *brca2* typically requires biallelic mutation or loss of at least one of these genes, and the specific combination of inherited mutations influences the development of LOH and the tumor phenotype. These results provide insight into cancer development associated with heritable *BRCA2* and *TP53* mutations.

## Introduction

Tumor suppressor genes provide a key barrier to neoplastic transformation by repressing survival and proliferation of abnormal cells. Germline mutations in the tumor suppressor genes *BRCA2* and *TP53* influence tumor susceptibility in many vertebrate species. In humans who inherit one mutated copy of *BRCA2* or *TP53,* tumor development is often associated with loss of the wildtype allele, indicating that somatic loss of heterozygosity (LOH) is important for neoplastic transformation [1]–[4]. However, tumors can occur in human carriers of *BRCA2* or *TP53* mutations without somatic LOH, suggesting that haploinsufficiency for either gene can lead to tumorigenesis [1], [5], [6].

*BRCA2* mutation in humans is associated with two distinct cancer susceptibility syndromes. Individuals who inherit one mutant allele for *BRCA2* experience an increased risk for breast and ovarian cancer in adulthood [7], [8], while individuals who inherit biallelic *BRCA2* mutations have a high incidence of malignancies during childhood [9]. *TP53* mutation may have a synergistic effect on tumorigenesis in *BRCA2*-associated cancers. Breast and ovarian cancers from *BRCA2*-heterozygous patients often develop *TP53* mutations [10]–[12], which may precede loss of the wildtype *BRCA2* allele [13], and are not attributable to a generalized or random increase in genetic mutations [10]. Similarly, tumor development is enhanced in *Brca2*-mutant mice and zebrafish with concomitant homozygous *Tp53* mutation or loss [14]–[17].

Although *TP53* dysfunction is important in *BRCA2*-associated carcinogenesis, the effect of coincident disruptions in these genes on tumorigenesis is not well defined. In this study, we investigated the relationship between inherited mutations in *brca2* and *tp53,* and somatic LOH for these genes, in tumorigenesis in zebrafish [16], [18]. Zebrafish with heterozygous *tp53^M214K^* mutation displayed a high incidence of malignant tumors. LOH for *tp53* was an important factor in malignant tumors from *brca2* wildtype and *brca2^Q658X^* heterozygous zebrafish, but was less important in *brca2^Q658X^* homozygous mutant zebrafish. Furthermore, the *brca2* mutation status influenced the age at tumor onset, tumor number, and tumor type. Lastly, malignant peripheral nerve sheath tumors consistently exhibited LOH for *tp53*, while undifferentiated sarcomas more commonly exhibited loss of *brca2* via homozygous mutation. These findings indicate that mutations in *brca2* and *tp53* enhance tumorigenesis in zebrafish, as seen in humans, and that the incidence and type of tumor depends upon the particular combination of mutations.

## Results

### Homozygous *brca2^Q658X^* mutation enhances tumor development in *tp53^M214K^* heterozygous zebrafish

We previously reported that homozygous *tp53^M214K^* mutation [18], combined with heterozygous or homozygous *brca2^Q658X^* mutation, accelerates tumorigenesis in zebrafish [16]. The *tp53^M214K^* mutation is a missense mutation [18] and the *brca2^Q658X^* mutation is a nonsense mutation [16] (mutant alleles subsequently designated as ‘*m*’). Here we describe tumor development in zebrafish that are wildtype (*brca2*+/+), heterozygous (*brca2+/m*), or homozygous (*brca2 m/m*) for the *brca2^Q658X^* mutation, on a heterozygous *tp53^M214K^* mutant background (*tp53+/m*).

**Figure 1:**
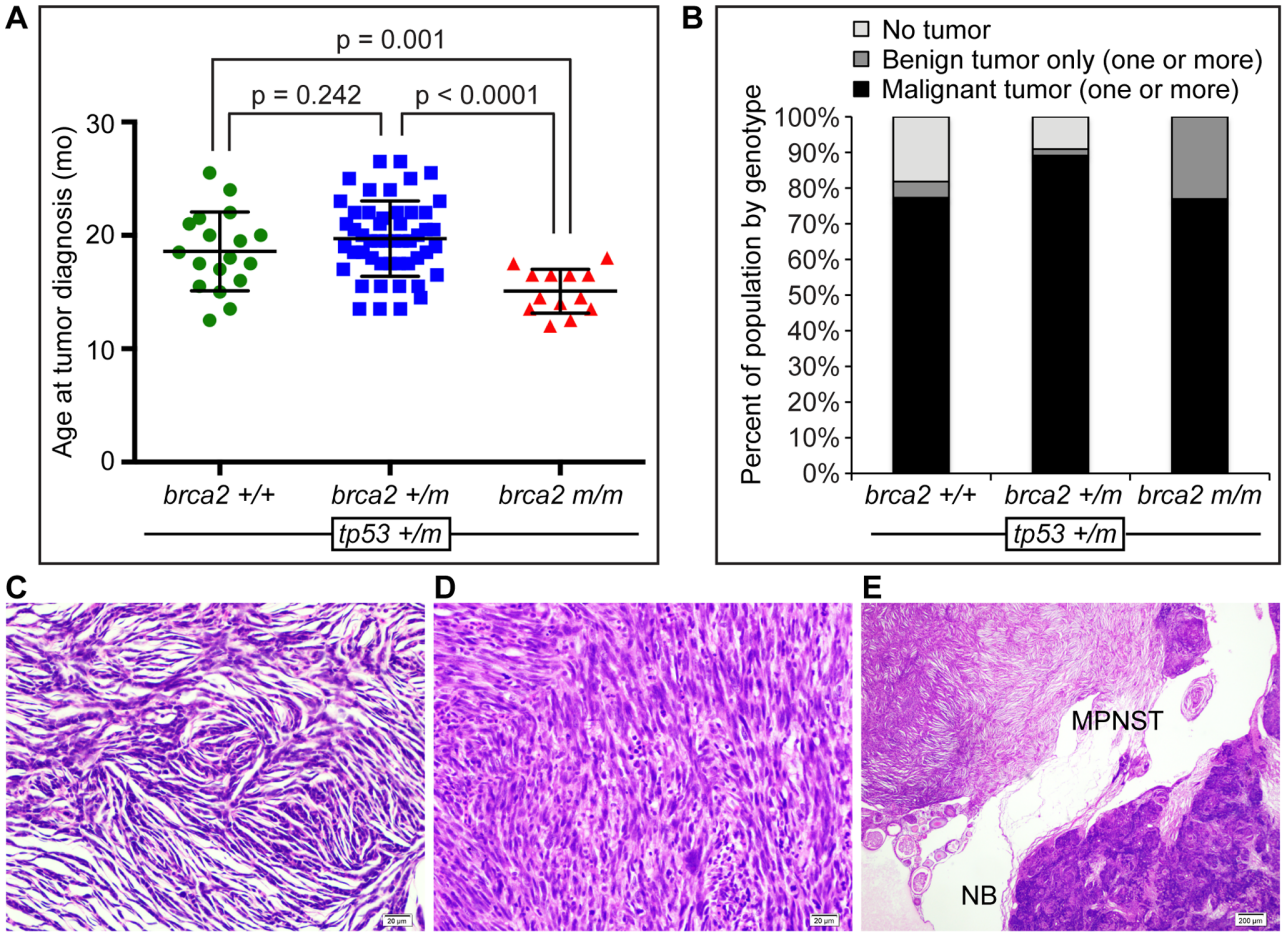
Tumor development *in tp53+/m* zebrafish is influenced by *brca2* mutation status. (**A**) Age at tumor diagnosis is significantly lower in *brca2 m/m*;*tp53+/m* zebrafish compared to *brca2*+/+;*tp53+/m* and *brca2+/m*;*tp53+/m* zebrafish. (**B**) The percentage of zebrafish that developed at least one malignant tumor, only benign tumors, or no tumors, in *brca2*+/+;*tp53+/m*, *brca2+/m*;*tp53+/m*, and *brca2 m/m*;*tp53+/m* zebrafish. (**C**) MPNST from a *brca2*+/+;*tp53+/m* zebrafish. (**D**) Undifferentiated sarcoma from a *brca2 m/m*;*tp53+/m* zebrafish. (**E**) MPNST and nephroblastoma from a *brca2+/m*;*tp53+/m* zebrafish. MPNST, malignant peripheral nerve sheath tumor; NB, nephroblastoma. Scale bars, 20 μm (C,D) and 200 μm (E).

We analyzed 90 *tp53+/m* zebrafish by histology and determined that the overall tumor incidence was 82% in *brca2+/+;tp53+/m* zebrafish, 91% in *brca2+/m;tp53+/m* zebrafish, and 100% in *brca2 m/m;tp53+/m* zebrafish (Table 1). There was no significant difference in tumor development between male and female zebrafish (Table 1). Tumor development occurred between 12.0 and 26.5 months of age (Figure 1A). The mean age at tumor diagnosis was statistically significantly lower in *brca2 m/m*;*tp53+/m* zebrafish compared to *brca2*+/+;*tp53+/m* or *brca2+/m*;*tp53+/m* zebrafish (Table 1 and Figure 1A). The mean age at tumor diagnosis was not significantly different between *brca2+/+;tp53+/m* and *brca2+/m;tp53+/m* zebrafish (Figure 1A). The overall survival for *brca2 m/m;tp53+/m* zebrafish declined rapidly compared to *brca2+/+;tp53+/m* and *brca2+/m;tp53+/m* zebrafish (Figure S1).

**Table 1:** Characteristics of tumor development in *brca2+/+;tp53+/m*, *brca2+/m;tp53+/m*, and *brca2 m/m;tp53+/m* zebrafish.

Tumor incidence in *tp53+/m* zebrafish			
	*brca2+/+*	*brca2+/m*	*brca2 m/m*
Number of animals analyzed	22	55	13
Animals with tumors	18 (82%)	50 (91%)	13 (100%)
* Male*	*6*	*16*	*12*
* Female*	*9*	*33*	*0*
* Sex not determined*	*3*	*1*	*1*
Animals without tumors	4 (18%)	5 (9%)	0 (0%)
* Male*	*1*	*3*	*0*
* Female*	*3*	*2*	*0*
* Sex not determined*	*0*	*0*	*0*
**Proportion of male and female animals with tumors in ** ***tp53+/m*** ** zebrafish**			
* brca2+/+;tp53+/m* males versus *brca2+/+;tp53+/m* females			p = 1.000
* brca2+/m;tp53+/m* males versus *brca2+/m;tp53+/m* females			p = 0.332
**Age at tumor diagnosis in ** ***tp53+/m*** ** zebrafish**			
	***brca2+/+***	***brca2+/m***	***brca2 m/m***
Mean age (mo)	18.6	19.7	15.1
**Characteristics of tumor development in ** ***tp53+/m*** ** zebrafish**			
	***brca2+/+***	***brca2+/m***	***brca2 m/m***
Number of animals analyzed	22	55	13
Malignant tumor (≥1)	17 (77%)	49 (89%)	10 (77%)
* Male*	*5*	*15*	*9*
* Female*	*9*	*33*	*0*
* Sex not determined*	*3*	*1*	*1*
Benign tumor only (≥1)	1 (5%)	1 (2%)	3 (23%)
* Male*	*1*	*1*	*3*
* Female*	*0*	*0*	*0*
* Sex not determined*	*0*	*0*	*0*
Animals with >1 tumor	4 (18%)	7 (13%)	7 (54%)
* Male*	*1*	*3*	*6*
* Female*	*3*	*4*	*0*
* Sex not determined*	*0*	*0*	*1*
**Proportion of animals with malignant tumors in ** ***tp53+/m*** ** zebrafish**			
* brca2+/+;tp53+/m* versus *brca2+/m;tp53+/m*			p = 0.277
* brca2+/+;tp53+/m* versus *brca2 m/m;tp53+/m*			p = 1.000
* brca2+/m;tp53+/m* versus *brca2 m/m;tp53+/m*			p = 0.358

To determine if the risk of malignant tumor development correlated with the *brca2* genotype in *tp53+/m* zebrafish, the numbers of zebrafish that developed at least one malignant tumor were determined for *brca2+/+;tp53+/m*, *brca2+/m;tp53+/m*, and *brca2 m/m;tp53+/m* cohorts (Table 1). Tumors with clear histologic evidence of tissue invasion and destruction were classified as malignant, while tumors that exhibited expansile but noninvasive growth were classified as benign. In all three cohorts, most tumor-bearing zebrafish developed malignant tumors, rather than benign tumors (Table 1 and Table 2). The proportion of zebrafish that developed at least one malignant tumor was not significantly different between any of the three cohorts (Figure 1B and Table 1). In comparison to the other cohorts, a greater proportion of *brca2 m/m;tp53+/m* zebrafish developed benign tumors (Figure 1B), due to an increased incidence of benign testicular tumors (Table 2).

**Table 2:** Tumor types developed by *brca2*+/+;*tp53*+/*m*, *brca2*+/*m*;*tp53*+/*m*, and *brca2* *m*/*m*;*tp53*+/*m* zebrafish.

Tumors developed by *tp53*+/m zebrafish^I^						
	*brca2*+*/*+		*brca2*+*/m*		*brca2 m/m*	
	n	Age (mo)	n	Age (mo)	n	Age (mo)
Malignant tumor type						
MPNST	11	16.0–25.5	30	13.5–26.5	1	14.5
Undifferentiated sarcoma	6	13.5–22.0	15	13.5–26.5	9	12.0–18.0
Nephroblastoma	0	−	4	16.5–20.5	0	−
Other malignant tumor	2	21.5–22.0	4	17.5–22.0	4	12.5–17.5
Benign tumor type						
Seminoma	2	12.5–25.5	4	21.0–25.5	4	13.5–16.5
Gonadal stromal tumor	0	−	0	−	3	12.0–16.5
Ultimobranchial adenoma	2	20.0–25.5	1	25.0	1	13.5
Total tumors	23	12.5–25.5	58	13.5–26.5	22	12.0–18.0

Abbreviations: MPNST, malignant peripheral nerve sheath tumor; mo, months.

I The total numbers of each tumor type observed by histologic analysis are reported. Some zebrafish developed more than one tumor (see Table 1).

The most common malignant tumor types observed in this study were malignant peripheral nerve sheath tumors (Figure 1C and Table 2) and undifferentiated sarcomas (Figure 1D and Table 2), the latter of which lacked sufficient histologic differentiation for more specific classification. Some zebrafish developed two or more histologically distinct and anatomically discrete tumors (Figure 1E). Over 50% of *brca2 m/m;tp53+/m* zebrafish developed more than one tumor; however, less than 20% of *brca2+/+;tp53+/m* and *brca2+/m;tp53+/m* zebrafish developed multiple tumors (Table 1). When multiple tumors occurred, they were distinguishable by histologic features, and were typically distinctly different tumor types (Figure 1E).

To determine the background level of tumor development over time in zebrafish with *brca2* mutation alone, we screened a small group of aged *brca2+/+;tp53+/+, brca2+/m;tp53+/+,* and *brca2 m/m;tp53+/+* zebrafish for tumor development (Table S1). The overall tumor incidence was higher in the *brca2 m/m;tp53+/+* cohort (Table S1 and Figure S2) compared to the other cohorts. This difference was attributable to an increased incidence of testicular tumors in *brca2 m/m;tp53+/+* zebrafish, as observed previously [16]. The development of multiple tumors in one animal was uncommon in all cohorts (Table S1).

### LOH for *brca2* and/or *tp53* is common in malignant zebrafish tumors

**Figure 2:**
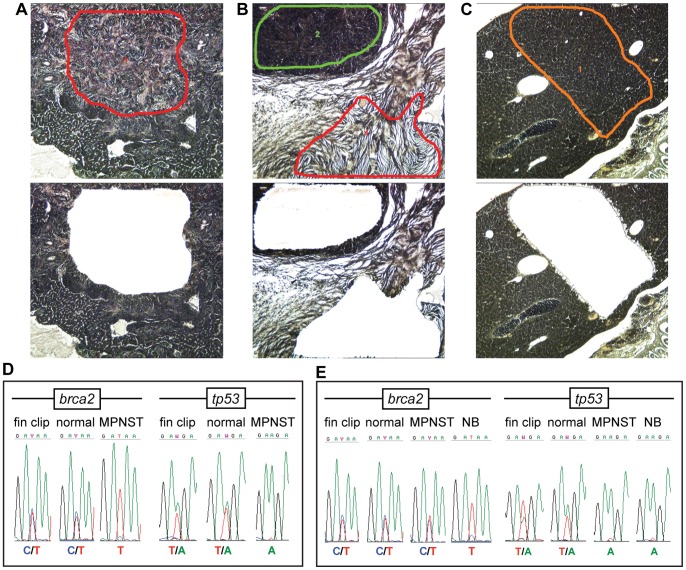
Malignant zebrafish tumors frequently develop LOH for *brca2* and/or *tp53*. (**A–C**), Before (upper panels) and after (lower panels) images of LCM-guided sample collection from an MPNST (A), an MPNST and a nephroblastoma (B), and a normal liver (C). Regions of sample collection are outlined in color. (**D**) MPNST from a *brca2+/m*;*tp53+/m* zebrafish shows loss of the *brca2* and *tp53* wildtype alleles. (**E**) MPNST and nephroblastoma from a *brca2+/m*;*tp53+/m* zebrafish show disparate LOH profiles. LOH, loss of heterozygosity; MPNST, malignant peripheral nerve sheath tumor; NB, nephroblastoma.

To identify LOH in zebrafish samples, we analyzed 31 histologically malignant tumors, four histologically benign tumors, and 28 matched normal tissue specimens from *tp53+/m* zebrafish that were *brca2*+/+, *brca2+/m*, or *brca2 m/m* (Table S2). Tissue samples were isolated by laser-capture microdissection (LCM) (Figures 2A–C) for DNA extraction, PCR amplification, and sequencing (Methods S1 and Table S3). Multiple tumors in individual zebrafish were isolated as separate samples (Figure 2B).

Sequence analyses revealed that LOH for *brca2* and *tp53* occurred frequently in malignant tumor specimens, but was not observed in normal tissues (Figures 2D and 2E and Table S2). Interestingly, different tumors analyzed from a single zebrafish did not necessarily exhibit the same LOH profile (Figure 2E and Table S2). Although *tp53* LOH always involved loss of the wildtype allele, two tumors from *brca2+/m*;*tp53+/m* zebrafish exhibited loss of the mutant *brca2* allele (Table S2). Loss of the mutant *BRCA2* allele has been reported in human tumors, but the functional significance of this change is unclear [6]. No LOH was detected in three of four histologically benign tumors or in any normal tissue specimens (Table S2).

### Malignant zebrafish tumors exhibit distinct LOH profiles that correlate with *brca2* genotype

**Figure 3:**
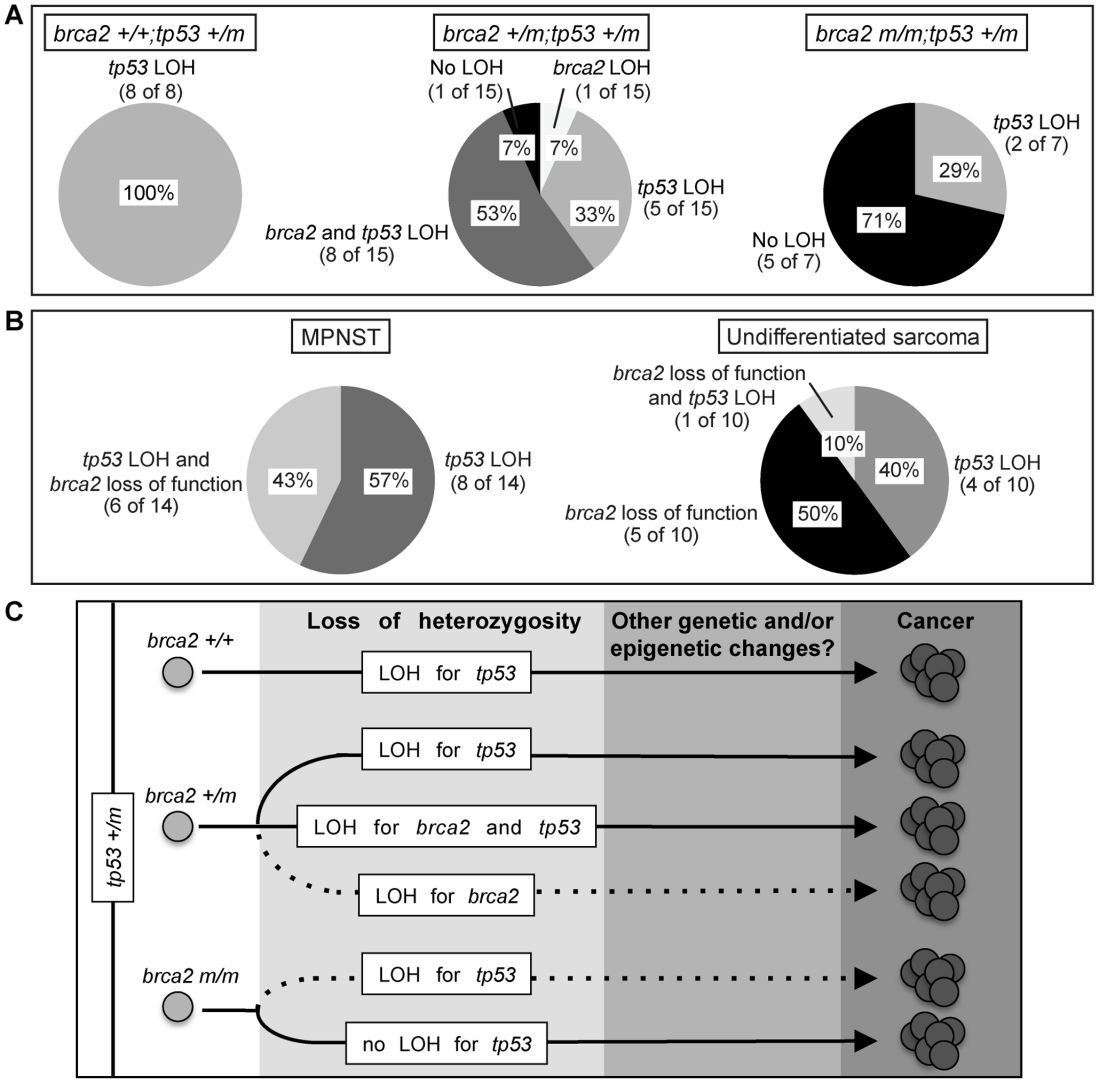
LOH profile and malignant tumor type correlate to *brca2* genotype. (**A**) Relative prevalence of each LOH profile for malignant tumors from *brca2+/+;tp53+/m*, *brca2+/m;tp53+/m*, and *brca2 m/m;tp53+/m* zebrafish. (**B**) LOH profiles of MPNST (left) and undifferentiated sarcomas (right). *brca2* loss of function refers to either homozygous *brca2* mutation or loss of the *brca2* wildtype allele. (**C**) Model depicting the roles for haploinsufficiency, LOH, and homozygous mutation in *brca2* and *tp53* during malignant transformation. Solid arrows indicate more common pathways of carcinogenesis; dashed arrows indicate less common pathways of carcinogenesis. MPNST, malignant peripheral nerve sheath tumor, LOH, loss of heterozygosity.

Segregation of malignant tumors by *brca2* genotype indicated that LOH status correlated with *brca2* mutation status (Figure 3A). In *brca2*+/+;*tp53+/m* zebrafish, *tp53* LOH occurred in 100% of malignant tumors (8 of 8). In *brca2+/m*;*tp53+/m* zebrafish, *tp53* LOH occurred in 86% of malignant tumors (13 of 15). Interestingly, over half of malignant tumors from *brca2+/m*;*tp53+/m* zebrafish developed LOH for both *brca2* and *tp53* (8 of 15, 53%). However, LOH for *brca2* alone was uncommon in this cohort (1 of 15, 7%). In *brca2 m/m*;*tp53+/m* zebrafish, *tp53* LOH occurred in only 29% of malignant tumors (2 of 7).

To investigate a second mechanism for *brca2* inactivation in malignant tumors from *brca2+/m*;*tp53+/m* zebrafish, we analyzed methylation status of the putative promoter region for *brca2* in four tumor specimens that retained the *brca2* wildtype allele. Significant methylation was not detected in tumor or matched control samples (Table S4).

### Malignant tumor type correlates to *brca2* genotype and LOH profile

Segregation of malignant tumors by tumor type indicated that *brca2* mutation status correlated with both tumor type and LOH profile (Table 3 and Figure 3B). These differences were observed for the two most common malignant tumor types observed in this study, malignant peripheral nerve sheath tumors (MPNST) and undifferentiated sarcoma. The proportion of zebrafish that developed MPNST was not significantly different between *brca2*+/+;*tp53*+/*m* and *brca2+/m*;*tp53+/m* cohorts (Table 3). However, the proportion of zebrafish that developed MPNST in *brca2 m/m*;*tp53+/m* zebrafish was significantly lower than both *brca2*+/+;*tp53*+/*m* and *brca2+/m*;*tp53+/m* cohorts (Table 3). The proportion of zebrafish that developed undifferentiated sarcomas in the *brca2 m/m*;*tp53+/m* cohort was higher than either the *brca2*+/+;*tp53*+/*m* or *brca2+/m*;*tp53+/m* cohorts, but this difference did not reach statistical significance (Table 3).

**Table 3:** Incidence of specific malignant tumor types in *tp53+/m* zebrafish.

Incidence of MPNST in *tp53+/m* zebrafish				
	*brca2*+*/*+^I^	*brca2*+*/m*		*brca2 m/m*
Number of animals compared	19	49		10
MPNST	11 (58%)	30 (61%)		1 (10%)
All other malignant tumor types	8 (42%)	19 (39%)		9 (90%)
Incidence of MPNST in *brca2+/+* versus *brca2+/m*			p = 1.000	
Incidence of MPNST in *brca2+/+* versus *brca2 m/m*			p = 0.019	
Incidence of MPNST in *brca2+/m* versus *brca2 m/m*			p = 0.004	
**Incidence of undifferentiated sarcoma in ** ***tp53+/m*** ** zebrafish**				
	***brca2*** **+** ***/*** **+** I	***brca2*** **+** ***/m***		***brca2 m/m***
Number of animals compared	19	49		10
Undifferentiated sarcoma	6 (32%)	14 (29%)		6 (60%)
All other malignant tumor types	13 (68%)	35 (71%)		4 (40%)
Incidence of undifferentiated sarcoma in *brca2+/+* versus *brca2+/m*			p = 1.000	
Incidence of undifferentiated sarcoma in *brca2+/+* versus *brca2 m/m*			p = 0.234	
Incidence of undifferentiated sarcoma in *brca2+/m* versus *brca2 m/m*			p = 0.074	

Abbreviations: MPNST, malignant peripheral nerve sheath tumor.

I One zebrafish in this group developed both an MPNST and an undifferentiated sarcoma, and was counted in both categories for each comparison.

MPNST and undifferentiated sarcomas were also correlated with different LOH profiles. All MPNST analyzed for LOH had lost the *tp53* wildtype allele, regardless of *brca2* status (14 of 14) (Figure 3B). Interestingly, this includes the single MPNST analyzed from the *brca2 m/m*;*tp53+/m* population, although *tp53* LOH was otherwise uncommon in tumors from this group. In comparison, 60% of undifferentiated sarcomas (6 of 10) had loss of the wildtype *brca2* allele, or occurred in *brca2 m/m*;*tp53+/m* fish, regardless of the *tp53* status (Figure 3B). However, this trend largely reflected the higher incidence of undifferentiated sarcomas in the *brca2 m/m*;*tp53+/m* cohort (Table 3).

## Discussion

*BRCA2* and *TP53* are well-known tumor suppressor genes that have been linked to defined human cancer syndromes, but the effect of combined genetic disruptions in these genes on carcinogenesis is not well defined. We examined tumor development in zebrafish with mutations in *brca2* and *tp53*, and describe the relationship between mutation status, development of somatic LOH, and development of malignant tumors.

In the absence of *tp53* mutation, tumor incidence was increased in *brca2 m/m;tp53+/+* zebrafish when compared to *brca2+/+;tp53+/+* and *brca2+/m;tp53+/+* zebrafish. However, on a *tp53+/m* background, zebrafish of all three *brca2* genotypes experienced similar tumor incidence. The age at tumor onset was statistically significantly lower, and the proportion of zebrafish with multiple tumors higher, in *brca2 m/m*;*tp53+/m* zebrafish when compared to *brca2+/+;tp53+/m* or *brca2+/m;tp53+/m* zebrafish. These results indicate that: 1) homozygous *brca2* mutation increases tumor development in zebrafish, similar to humans with germline biallellic *BRCA2* mutations, and 2) homozygous *brca2* mutation enhances carcinogenesis in *tp53+/m* zebrafish, supporting previous work that indicates a collaborative relationship between *BRCA2* and *TP53* in human carcinogenesis.

In humans, somatic LOH occurs frequently in tumors from patients who inherit one mutated copy of *BRCA2* or *TP53* [1]–[4]. To investigate the role for LOH in carcinogenesis in zebrafish with *brca2* and *tp53* mutations, we examined malignant zebrafish tumors and matched normal tissues for evidence of LOH. Loss of the wildtype alleles for *tp53* and *brca2* was common in malignant zebrafish tumors, implicating LOH as an important contributor to carcinogenesis in this species.

Importantly, development of LOH was dependent on *brca2* mutation status. Since all malignant tumors from *brca2+/+;tp53+/m* zebrafish developed *tp53* LOH, we conclude that LOH for *tp53* is necessary for carcinogenesis in *tp53+/m* zebrafish without *brca2* mutation. In contrast, the majority of malignant tumors from *brca2 m/m*;*tp53+/m* zebrafish did not develop somatic LOH for *tp53*. These findings suggest that on a *tp53+/m* background, the presence or absence of functional *brca2* may influence subsequent genetic alterations required for carcinogenesis.

While most malignant tumors from *brca2+/m;tp53+/m* zebrafish developed LOH for *tp53*, LOH for *brca2* was comparatively less common. These results suggest that in *brca2+/m*;*tp53+/m* zebrafish, biallelic inactivation or loss of *brca2* is either not required for tumorigenesis, occurs late in disease, or is achieved by other mechanisms. These possibilities have been previously postulated to explain why some cancers in humans with heterozygous *BRCA2* mutation do not develop *BRCA2* LOH or promoter methylation [1], [6]. In comparison, *TP53* mutation is thought to be a relatively early event in the pathogenesis of *BRCA2*-associated cancer in humans with heterozygous *BRCA2* mutation [13]. The high incidence of *tp53* LOH in malignant tumors from *brca2+/m;tp53+/m* zebrafish supports the concept that *TP53* dysfunction is a critical and potentially early step in *BRCA2*-associated carcinogenesis.

In addition to *brca2* genotype, LOH status also correlated with malignant tumor type. All MPNST evaluated for LOH had lost the *tp53* wildtype allele, regardless of *brca2* status. Several previous studies in zebrafish support an association between loss of functional *tp53* and MPNST [18]–[21]. In contrast, undifferentiated sarcomas were most commonly associated with homozygous *brca2* mutation. These patterns of gene disruptions may reflect disparate roles for *brca2* and *tp53* in zebrafish tumorigenesis. Interestingly, human cancer syndromes linked to heritable *BRCA2* mutations are also limited to a small range of tumor types [7]–[9].

The methods applied in this study represent a minimum estimate for LOH in malignant zebrafish tumors, as screening for LOH was done by sequencing within the exons containing the *brca2^Q658X^* and *tp53^M214K^* germline mutations. Other mechanisms of gene inactivation may also have contributed to carcinogenesis in this population. When we examined the methylation status of the putative *brca2* promoter in a small set of normal and tumor samples from *brca2+/m;tp53+/m* zebrafish, we did not find evidence of promoter methylation in any sample. While we cannot rule out the possibility that *brca2* promoter methylation contributed to tumorigenesis in this study, *BRCA2* promoter methylation occurs infrequently in tumor specimens from humans with heterozygous *BRCA2* mutations [1].

Human cancers display a remarkable degree of genetic heterogeneity [22], [23]. Both inter- and intratumoral heterogeneity are influenced by genetic factors, such as genomic instability, and nongenetic factors, such as tumor microenvironment [23], [24]. Two observations from the current study indicate a degree of heterogeneity among malignant zebrafish tumors. First, different tumors from the same animal did not necessarily exhibit the same LOH profile, suggesting that tumor initiation and/or progression could have involved different mechanisms, and may have been influenced by the site of onset and cell of origin. Second, a small number of tumors exhibited partial LOH for *brca2* or *tp53* (Table S2), indicating that a subset of tumor cells retained the wildtype allele for these genes. This suggests the presence of genetically diverse subclones within malignant zebrafish tumors, as has been observed in human cancers [22], [23]. Further investigation of genetic diversity in zebrafish tumors will be required to understand tumor heterogeneity in this species.

In this study, we demonstrate that LOH for *brca2* and *tp53* represents a conserved mechanism for carcinogenesis in zebrafish, and suggest that the relative importance of LOH or haploinsufficiency for *brca2* and *tp53* in driving carcinogenesis is dictated by *brca2* genotype (Figure 3C). In zebrafish that are wildtype or heterozygous for *brca2* mutation, *tp53* LOH appears to be a critical step in driving carcinogenesis. In contrast, cancers associated with homozygous *brca2* mutation do not required *tp53* LOH. We have previously reported accelerated tumorigenesis in *brca2+/m;tp53 m/m* and *brca2 m/m;tp53 m/m* zebrafish compared to *brca2+/+;tp53 m/m* zebrafish [16]. From these two studies, we conclude that carcinogenesis in zebrafish with germline mutations in *brca2* and *tp53* typically requires biallelic inactivation or loss of at least one of these two genes, and this effect is enhanced by haploinsufficiency or biallelic loss of the other gene.

The collaborative effects of *BRCA2* and *TP53* mutations on carcinogenesis have been previously described in human cancer. Genomic instability is thought to be a significant factor in human carcinogenesis [22], [23], and germline *BRCA2* mutation is linked to an increased mutation rate in *Brca2*-mutant mice [25] and in *BRCA2*-associated human cancer [26], [27]. Coincident or subsequent *TP53* pathway disruption in *BRCA2*-deficient cells may permit survival and proliferation of cell populations with significant genetic aberrations, ultimately leading to neoplastic transformation. Further investigation of genetic and/or epigenetic alterations accompanying *brca2* mutation in malignant zebrafish tumors may uncover additional factors that contribute to *BRCA2*-associated cancer in humans.

## Materials and Methods

### Ethics statement

Zebrafish were monitored for clinical and gross evidence of tumor development and humanely euthanized with 50X Tricaine in system water buffered with Sodium Bicarbonate (0.7 grams/liter). All animal studies were approved by the Intramural Animal Care and Use Committee, National Cancer Institute, National Institutes of Health, Bethesda, MD (Animal Study Protocol #MB-081).

### Zebrafish maintenance

Experiments were performed with adult zebrafish from the *brca2^hg5^* and *tp53^zdf1^* mutant zebrafish lines carrying the *brca2^Q658X^* [16] and *tp53^M214K^* [18] mutations, respectively. All zebrafish evaluated in this study were related. For additional details, see Methods S1.

### Histologic analyses

Zebrafish were processed for histology as previously described [16]. Tumors were classified based on histologic features, and the number of histologically and anatomically distinct tumors was determined for each specimen. Histologic diagnoses were made without knowledge of the *brca2* genotype or loss of heterozygosity status. For additional details, see Methods S1.

### LCM and DNA extraction from paraffin-embedded zebrafish tissues

Tissue sections were placed on PEN-membrane glass slides, and tumor and normal tissue specimens were individually collected by laser-capture microdissection. Collected specimens were routinely processed for DNA isolation (See Methods S1).

### LOH analyses

LOH analyses were achieved by PCR amplification and sequencing over the *brca2^Q658X^* and *tp53^M214K^* mutation sites. See Methods S1, Figure S3, and Table S3 for details.

### CpG island identification and pyrosequencing methylation detection assay

The zebrafish *brca2* promoter has not been characterized, but a CpG island as determined by NCBI algorithm is predicted to occur 306 base pairs upstream of the 5′ position of the translational start codon for *brca2* on chromosome 15 (*Danio rerio* genome version Zv9). DNA isolated from LCM-collected samples was routinely processed for bisulfite conversion, PCR amplification, and Pyrosequencing analysis for methylation status of this CpG island (See Methods S1).

### Statistics

Data sets comprised of the proportions of male or female animals that developed tumors were compared by Fisher’s exact test (GraphPad Prism, version 6.0b), and P<0.05 was accepted to indicate statistical significance. Data sets comprised of age at tumor diagnosis were compared by unpaired t-test with Welch’s correction (GraphPad Prism, version 6.0b), and P<0.05 was accepted to indicate statistical significance. Data sets comprised of the proportions of animals with malignant tumors were compared by Fisher’s exact test with Mehta’s modification (GraphPad Prism, version 6.0b), and P<0.05 was accepted to indicate statistical significance. For additional details on statistical comparisons, see Methods S1.

## Supporting Information

Figure S1 **Overall survival declines rapidly in** ***brca2 m/m;tp53+/m*** **zebrafish.** Kaplan-Meier survival curves for all *tp53+/m* zebrafish described in this study show that the survival curve for the *brca2 m/m;tp53+/m* cohort declined rapidly in comparison to *brca2+/+;tp53+/m* and *brca2+/m;tp53+/m* cohorts.
(TIF)

Figure S2 **Tumorigenesis is enhanced by** ***brca2*** **mutation.** The percentage of zebrafish that developed tumors (benign or malignant) is higher in the *brca2 m/m;tp53+/+* cohort than in *brca2+/+;tp53+/+* or *brca2+/m;tp53+/m* cohorts.
(TIF)

Figure S3 **Diagram of zebrafish** ***brca2*** **and** ***tp53*** **genes indicating mutation and SNP positions relevant to LOH analyses.** (**A**) Diagram of zebrafish *brca2* indicating the locations of the *brca2^Q658X^* mutation, and the locations of single nucleotide polymorphisms (SNPs) used to distinguish wildtype and mutant alleles. (**B**) Diagram of zebrafish *tp53* indicating the locations of the *tp53^M214K^* mutation, and the locations of SNPs used to distinguish wildtype and mutant alleles. Vertical lines indicate PCR primer positions.
(TIF)

Table S1 **Characteristics of tumor development in** ***brca2+/+;tp53+/+*** **,** ***brca2+/m;tp53+/+*** **, and** ***brca2 m/m;tp53+/+*** **zebrafish.**
(DOC)

Table S2 **Summary of LOH analyses performed on tumor specimens and matched normal tissue specimens collected from** ***brca2 +/+;tp53 +/m*** **,** ***brca2 +/m;tp53 +/m*** **, and** ***brca2 m/m;tp53 +/m*** **zebrafish.**
(PDF)

Table S3 **Summary of target and primer sequences used for LOH analyses of normal and tumor specimens.**
(DOC)

Table S4 **Summary of CpG analyses from normal and tumor specimens from** ***brca2+/m;tp53+/m*** **zebrafish.**
(DOC)

Methods S1 (DOC)
