# Supplementary figures and images for: brca2 and tp53 Collaborate in Tumorigenesis in Zebrafish
Source: PLoS One. 2014 Jan 29;9(1):e87177. doi: 10.1371/journal.pone.0087177 (PMC3906131; doi:10.1371/journal.pone.0087177)

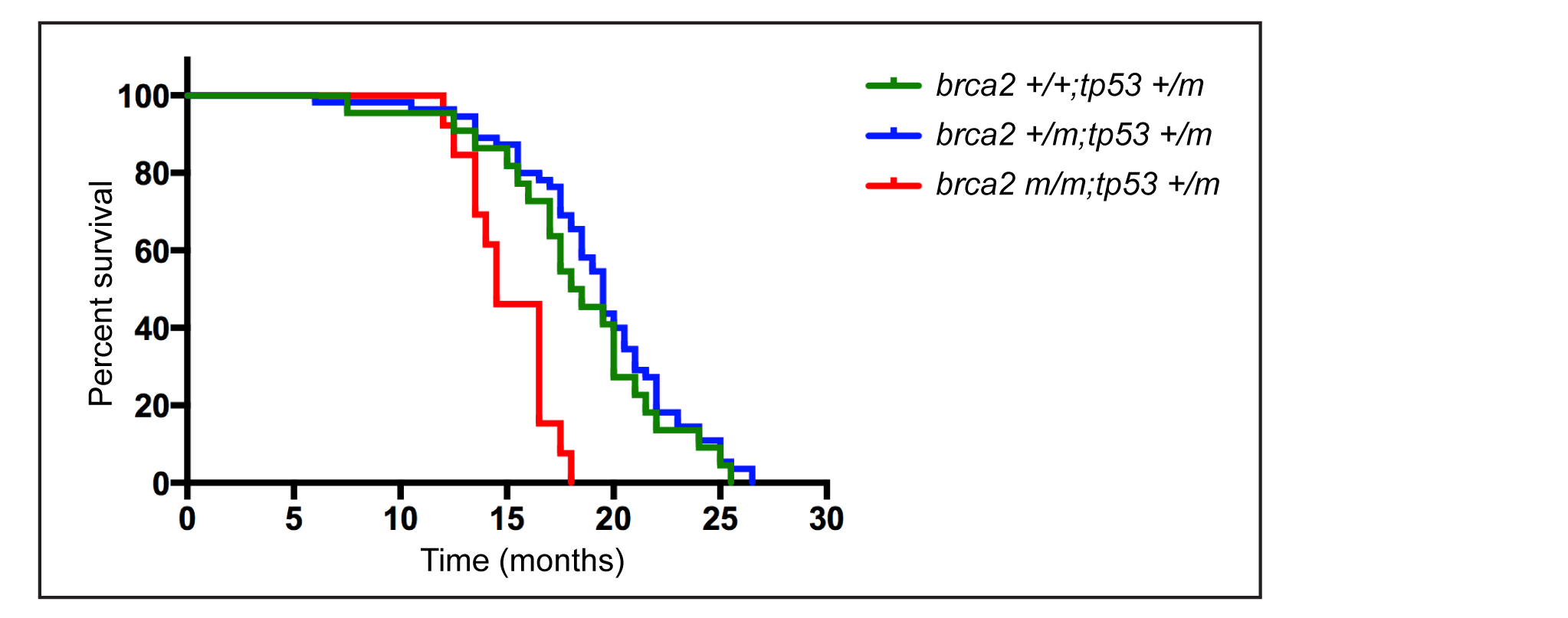

Supplement: Figure S1 — Overall survival declines rapidly in brca2 m/m;tp53+/m zebrafish. Kaplan-Meier survival curves for all tp53+/m zebrafish described in this study show that the survival curve for the brca2 m/m;tp53+/m cohort declined rapidly in comparison to brca2+/+;tp53+/m and brca2+/m;tp53+/m cohorts. (TIF) [file pone.0087177.s001.tif]

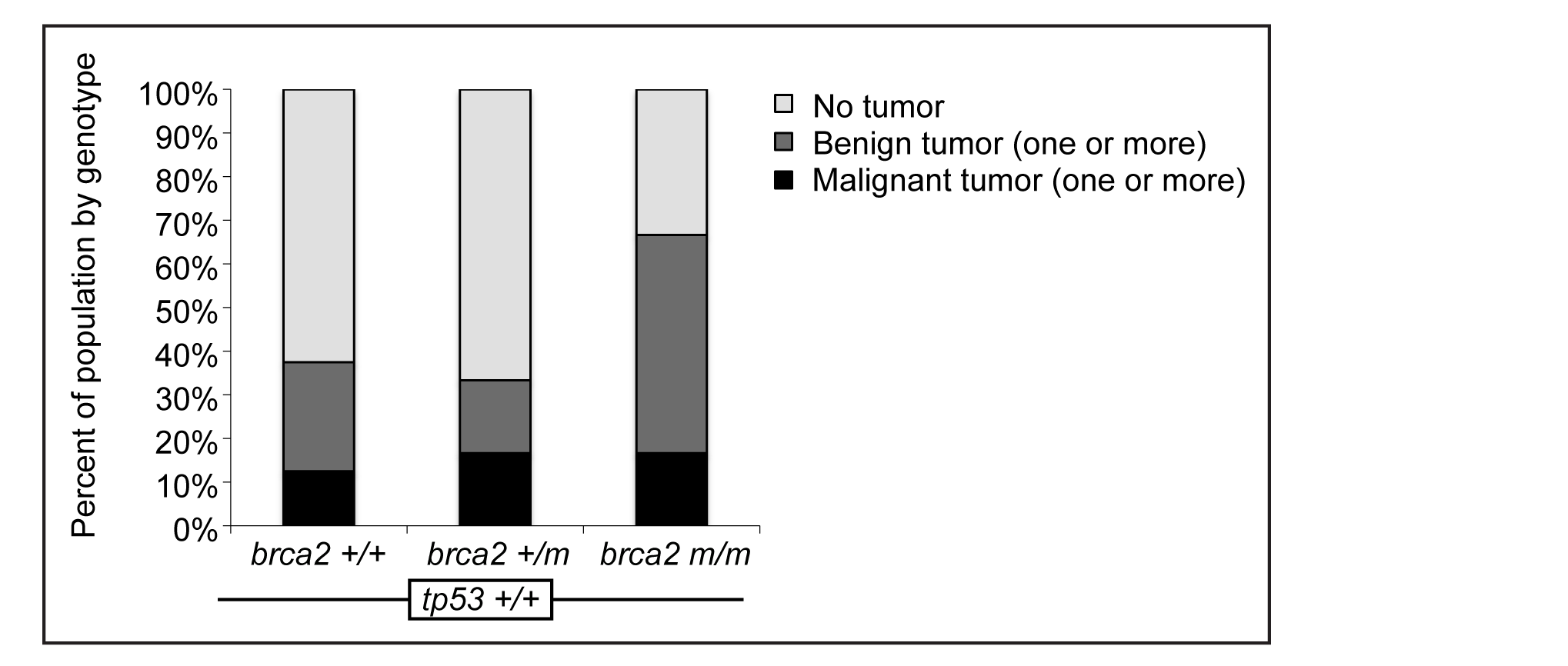

Supplement: Figure S2 — Tumorigenesis is enhanced by brca2 mutation. The percentage of zebrafish that developed tumors (benign or malignant) is higher in the brca2 m/m;tp53+/+ cohort than in brca2+/+;tp53+/+ or brca2+/m;tp53+/m cohorts. (TIF) [file pone.0087177.s002.tif]

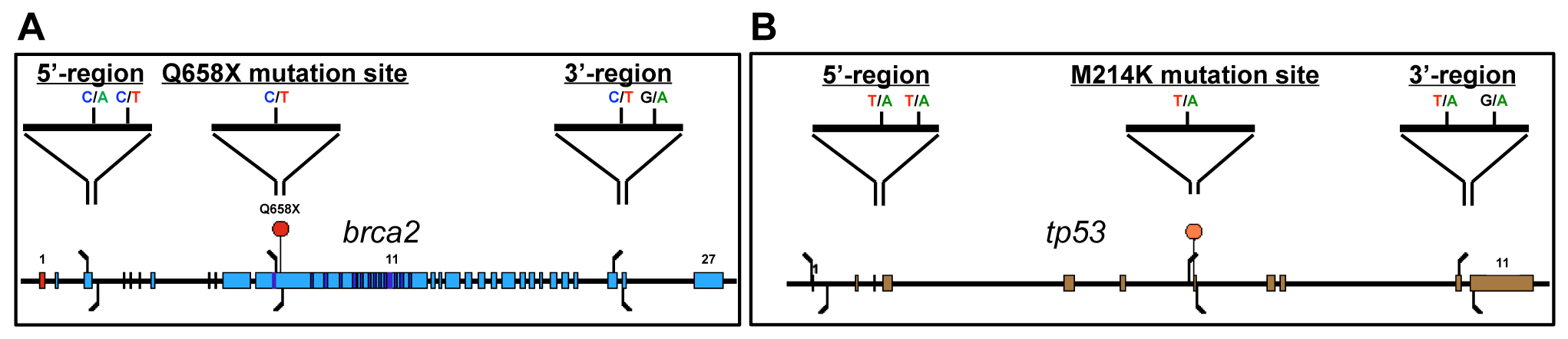

Supplement: Figure S3 — Diagram of zebrafish brca2 and tp53 genes indicating mutation and SNP positions relevant to LOH analyses. (A) Diagram of zebrafish brca2 indicating the locations of the brca2Q658X mutation, and the locations of single nucleotide polymorphisms (SNPs) used to distinguish wildtype and mutant alleles. (B) Diagram of zebrafish tp53 indicating the locations of the tp53M214K mutation, and the locations of SNPs used to distinguish wildtype and mutant alleles. Vertical lines indicate PCR primer positions. (TIF) [file pone.0087177.s003.tif]
