# Supplementary material for: brca2 and tp53 Collaborate in Tumorigenesis in Zebrafish
Source: PLoS One. 2014 Jan 29;9(1):e87177. doi: 10.1371/journal.pone.0087177 (PMC3906131; doi:10.1371/journal.pone.0087177)
Supplement: Table S1 — Characteristics of tumor development in brca2+/+;tp53+/+ , brca2+/m;tp53+/+ , and brca2 m/m;tp53+/+ zebrafish. (DOC) [file pone.0087177.s004.doc]

| **Tumor incidence in *tp53 +/+* zebrafish** | | | | | | | | | | | | | |
| --- | --- | --- | --- | --- | --- | --- | --- | --- | --- | --- | --- | --- | --- |
|  | | | | | | *brca2 +/+* | | | | *brca2 +/m* | | *brca2 m/m*^I^ | |
| Number of animals analyzed | | | | | | 8 | | | | 6 | | 12 | |
|  | Animals with tumors | | | | | 3 (38%) | | | | 2 (33%) | | 8 (67%) | |
|  | | | *Male* | | | *2* | | | | *1* | | *8* | |
|  | | | *Female* | | | *1* | | | | *1* | | *0* | |
|  | Animals without tumors | | | | | 5 (62%) | | | | 4 (67%) | | 4 (33%) | |
|  | | | *Male* | | | *1* | | | | *1* | | *3* | |
|  | | | *Female* | | | *5* | | | | *3* | | *0* | |
|  | | | *Sex not determined* | | | *0* | | | | *0* | | *1* | |
| **Characteristics of tumor development in *tp53 +/+*  zebrafish** | | | | | | | | | | | | | |
|  | | | | | | *brca2 +/+* | | | | *brca2 +/m* | | *brca2 m/m* | |
| Number of animals analyzed | | | | | | 8 | | | | 6 | | 12 | |
|  | | Malignant tumor (1 or more) | | | | 1 (13%) | | | | 1 (17%) | | 2 (17%) | |
|  | | Benign tumor only (1 or more) | | | | 2 (25%) | | | | 1 (17%) | | 6 (50%) | |
| Animals with > 1 tumor | | | | | | 0 (0%) | | | | 0 (0%) | | 0 (0%) | |
| **Tumors developed by *tp53* +/+ zebrafish** | | | | | | | | | | | | | |
|  | | | | *brca2 +/+* | | | | *brca2 +/m* | | | *brca2 m/m* | | |
|  | | | | n | Age (mo) | | | n | Age (mo) | | n | | Age (mo) |
| Malignant tumor type | | | |  | | |  | | | |  | | |
|  | MPNST | | | 0 | − | | | 0 | − | | 1 | | 23.5 |
|  | Undifferentiated sarcoma | | | 1 | 27.0 | | | 0 | − | | 0 | | − |
|  | Other malignant tumor | | | 0 | − | | | 1 | 27.0 | | 1 | | 24.5 |
| Benign tumor type | | | |  | | | |  | | |  | | |
|  | | Seminoma | | 2 | 27.0 | | | 1 | 27.0 | | 5 | | 22.0 – 27.0 |
|  | | Gonadal stromal tumor | | 0 | − | | | 0 | − | | 1 | | 27.0 |
| Total tumors | | | | 3 | 27.0 | | | 2 | 27.0 | | 8 | | 22.0 – 27.0 |

**Table S1** Characteristics of tumor development in *brca2 +/+;tp53 +/+*, *brca2 +/m;tp53 +/+*, and *brca2 m/m;tp53 +/+* zebrafish.
