# Supplementary material for: brca2 and tp53 Collaborate in Tumorigenesis in Zebrafish
Source: PLoS One. 2014 Jan 29;9(1):e87177. doi: 10.1371/journal.pone.0087177 (PMC3906131; doi:10.1371/journal.pone.0087177)
Supplement: Table S2 — Summary of LOH analyses performed on tumor specimens and matched normal tissue specimens collected from brca2 +/+;tp53 +/m , brca2 +/m;tp53 +/m , and brca2 m/m;tp53 +/m zebrafish. (PDF) [file pone.0087177.s005.pdf]

Table S2 Summary of LOH analyses performed on tumor specimens and matched normal tissue specimens collected from *brca2 +/+; tp53 +/m*, *brca2 +/m;tp53 +/m*, and *brca2 m/m;tp53 +/m* zebrafish.

					**Loss of Heterozygosity**			
	**Specimen**	**Age (mo)**	**Sex**	**Tissue specimen**	***brca2* allele**		***tp53* allele**	
					**wildtype**	**mutant**	**wildtype**	**mutant**
***brca2* +/+**	1	17.0	M	1) MPNST	–	–	Y(p)	N
				2) Normal	–	–	N	N
	2	17.5	UD	1) Undifferentiated sarcoma	–	–	Y	N
				2) Normal	–	–	N	N
	3	18.0	M	1) MPNST	–	–	Y	N
				2) Normal	–	–	N	N
	4	18.5	F	1) MPNST	–	–	Y(p)	N
				2) Normal	–	–	N	N
	5	21.0	M	1) Undifferentiated sarcoma	–	–	Y	N
				2) Normal	–	–	N	N
	6	22.0	M	1) Undifferentiated sarcoma	–	–	Y	N
				2) Malignant seminoma	–	–	Y	N
				3) Normal	–	–	N	N
	7	24.0	F	1) MPNST	–	–	Y	N
				2) Normal	–	–	N	N
	8	18.0	F	1) MPNST	N	N	Y	N
				2) Normal	N	N	N	N
	9	18.5	F	1) MPNST	Y	N	Y	N
				2) Normal	N	N	N	N
	10	19.5	F	1) Rhabdomyosarcoma	N	N	Y(p)	N
				2) Normal	N	N	N	N
	11	19.5	F	1) MPNST	Y (p)	N	Y	N
				2) Normal	N	N	N	N
	12	19.5	F	1) MPNST	N	N	Y	N
				2) Nephroblastoma	Y	N	Y	N

brca2 +/m				3) Normal	N	N	N	N
	13	20.5	F	1) Nephroblastoma	N	Y	Y	N
				2) Normal	N	N	N	N
	14	21.0	M	1) Undifferentiated sarcoma (ventral coelom)	Y	N	Y(p)	N
				2) Undifferentiated sarcoma (ventral coelom)	N	N	N	N
				3) Undifferentiated sarcoma (dorsal coelom/body wall)	Y	N	N	N
				4) Normal	N	N	N	N
	15	21.0	M	1) Hemangiosarcoma	N	N	N	N
				2) Seminoma	N	N	N	N
				3) Normal	N	N	N	N
	16	21.5	F	1) MPNST	N	N	Y(p)	N
				2) Normal	N	N	N	N
	17	22.0	M	1) MPNST	N	N	Y	N
				2) Normal	N	N	N	N
	18	23.0	F	1) MPNST	Y	N	Y(p)	N
				2) Normal	N	N	N	N
	19	24.0	M	1) MPNST	Y	N	Y	N
				2) Normal	N	N	N	N
	20	24.0	M	1) Undifferentiated sarcoma	N	Y	Y	N
				2) Normal	N	N	N	N
	21	25.0	M	1) Seminoma	N	N	N	N
				2) Normal	N	N	N	N
	22	25.0	M	1) MPNST	Y	N	Y	N
				2) Seminoma	N	N	N	N
				3) Ultimobranchial adenoma	–	–	–	–
				4) Normal	N	N	N	N
	23	14.0	M	1) Undifferentiated sarcoma	–	–	Y	N
				2) Normal	–	–	N	N
	24	14.5	M	1) MPNST	–	–	Y	N
				2) Gonadal stromal tumor	–	–	–	–

brca2 m/m				3) Seminoma	–	–	–	–
				4) Normal	–	–	N	N
	25	14.5	UD	1) Undifferentiated sarcoma (gonad)	–	–	N	N
				2) Undifferentiated sarcoma (dorsal coelom/body wall)	–	–	–	–
				3) Normal	–	–	N	N
	26	16.5	M	1) Undifferentiated sarcoma (retrobulbar/intraocular)	–	–	N	N
				2) Undifferentiated sarcoma (gonad)	–	–	N	N
				3) Normal	–	–	N	N
	27	16.5	M	1) Seminoma	–	–	Y	N
				2) Undifferentiated sarcoma (coelom)	–	–	N	N
				3) Undifferentiated sarcoma (tail)	–	–	–	–
				4) Normal	–	–	N	N
	28	18.0	M	1) Undifferentiated sarcoma	–	–	N	N
				2) Normal	–	–	N	N

Abbreviations: Mo, months, F, female, M, male, ND, sex not determined, MPNST, malignant peripheral nerve sheath tumor, Y, loss of the allele, N, no loss of the allele, −, not performed, Y(p), partial loss of the allele.

[A] This undifferentiated sarcoma was collected as two samples, with intratumoral pigmented cells (#1) and without intratumoral pigmented cells (#2). As this tumor exhibited a complex LOH profile resulting from tissue heterogeneity, it was excluded from further analyses.
