# Supplementary material for: brca2 and tp53 Collaborate in Tumorigenesis in Zebrafish
Source: PLoS One. 2014 Jan 29;9(1):e87177. doi: 10.1371/journal.pone.0087177 (PMC3906131; doi:10.1371/journal.pone.0087177)
Supplement: Table S3 — Summary of target and primer sequences used for LOH analyses of normal and tumor specimens. (DOC) [file pone.0087177.s006.doc]

**Table S3** Summary of target and primer sequences used for LOH analyses of normal and tumor specimens.

| ***brca2* target and primer sequences for LOH analyses** | | |
| --- | --- | --- |
| **Analysis site** | **Parameter** | **Sequence**^I^ |
| *brca2^Q658X^* mutation | Target | ccctgtagga**C/T**aatccatccc |
|  | 5’ primers | (#64) CATTAGATGAAGCTGCAGGAGATGG  (#75) GTTTCAAGAAGCATTCCTGCACC |
|  | 3’ primers | (#45) GTCTTGGAAGCATCACTAACACTCAC  (#74) CATTTTAAACCCCTTCGGACTGTCAG |
| 5’ SNPs | Target | gaaaagtcac**C/A**aaatgaaggtgtcgttggtgttaagttatgtgttaagtatgtgtatgtaaaa**C/T**tgaggtaaag |
|  | 5’ primer | (#69) GAGAGCATCAAAGCACCAGAAAC |
|  | 3’ primer | (#40) CAGACCGAATATTCAAGTACAACATAGGGAC |
| 3’ SNPs | Target | aagaatctta**C/T**ctgcaagaagctatggctcatctgaagac**A/G**tttgcacaggttac |
|  | 5’ primer | (#65) GAGTGCTATCTGGGCCTGTGCC |
|  | 3’ primer | (#66) CCACATCTGACACCTTCTCCTCC |
| ***tp53* target and primer sequences for LOH analyses** | | |
| **Analysis site** | **Parameter** | **Sequence** |
| *tp53^M214K^* mutation | Target | atggggggga**T/A**gaaccgcagg |
|  | 5’ primers | (#F2) TCCTGTTTTTGCAGCTTGGTG  (#F5) CCATCTGTTTAACAGTCACATTTTCCTG |
|  | 3’ primers | (#R4) CAGTTCACAAGAGGAGGAATCAAATATGC  (#R6) CAGAGTGATGATTGTGAGGATGGG |
| 5’ SNPs | Target | agttctttgt**T/A**tgagcttcaacagattaatactaatttctctctcttccttt**T/A**caattgtctc |
|  | 5’ primer | (#P53-C) GGAGCTCTGGGAGAAGAATTTGATG |
|  | 3’ primer | (#P53-D) GATGTCCCAGCAAGAGCCACC |
| 3’ SNPs | Target | tatccaattt**T/A**tttaat**G/A**caacacctgt |
|  | 5’ primer | (#P53-G) GATGCTGAAAAGTATCGTCAGAAATTGTAAG |
|  | 3’ primer | (#P53-H) CTTCGTCCTTCACCATCAGCTTC |

^I^ Capitalized and bolded nucleotides represent the point mutation or SNP(s) found in each target sequence.
