# Supplementary material for: brca2 and tp53 Collaborate in Tumorigenesis in Zebrafish
Source: PLoS One. 2014 Jan 29;9(1):e87177. doi: 10.1371/journal.pone.0087177 (PMC3906131; doi:10.1371/journal.pone.0087177)
Supplement: Table S4 — Summary of CpG analyses from normal and tumor specimens from brca2+/m;tp53+/m zebrafish. (DOC) [file pone.0087177.s007.doc]

**Table S4** Summary of CpG analyses from normal and tumor specimens from *brca2 +/m;tp53 +/m* zebrafish.

| **Specimen characteristics** | | | | **Tumor LOH status** | | **CpG analysis** | |
| --- | --- | --- | --- | --- | --- | --- | --- |
| ***brca2 +/m*** | **Specimen**^I^ | **Age (mo)** | **Tumor** | ***brca2*** | ***tp53*** | **Normal** | **Tumor** |
|  | 8 | 18.0 | MPNST | None | Lost wildtype allele | − | NS |
|  | 10 | 19.5 | Rhabdomyosarcoma | None | Lost wildtype allele | NS | − |
|  | 13 | 20.5 | Nephroblastoma | Lost mutant allele | Lost wildtype allele | NS | NS |
|  | 20 | 24.0 | Undifferentiated sarcoma | Lost mutant allele | Lost wildtype allele | NS | NS |

Abbreviations: Mo, months, F, female, M, male, MPNST, malignant peripheral nerve sheath tumor; NS, not significant; −, PCR amplification failure in multiple runs.

^I^ Specimen number refers to the specimen number assigned to each individual in Table S3.
