# Supplementary material for: brca2 and tp53 Collaborate in Tumorigenesis in Zebrafish
Source: PLoS One. 2014 Jan 29;9(1):e87177. doi: 10.1371/journal.pone.0087177 (PMC3906131; doi:10.1371/journal.pone.0087177)
Supplement: Methods S1 — (DOC) [file pone.0087177.s008.doc]

**Methods S1**

**Tumor screening in *tp53 +/m* zebrafish.** All *tp53 +/m* zebrafish in this study were from two generations: first, a population of *brca2 +/+;tp53 +/m* and *brca2 +/m;tp53 +/m* siblings, and second, the offspring from an incross of the *brca2 +/m;tp53 +/m* zebrafish. Zebrafish were monitored for clinical and gross evidence of tumor development, and zebrafish that were humanely euthanized or found dead were processed for histology as described . Specimens were typically sectioned sagitally, and one to five hematoxylin and eosin stained sections were evaluated for each zebrafish specimen. Sexual identity was confirmed by histologic examination of ovaries or testes; if gonads were not identifiable by histology, zebrafish were categorized as ‘sex not determined’.

**Tumor screening in *tp53 +/+* zebrafish**. The *tp53 +/+* cohort was comprised of 26 *brca2 +/+,* *brca2 +/m*, and *brca2 m/m* zebrafish that were siblings to the *tp53 +/m* cohort described above. Eight *tp53 +/+* zebrafish died or were euthanized during the study (*brca2 +/+;tp53 +/+*, n = 2, and *brca2 m/m;tp53 +/+*, n = 6). 18 zebrafish were collected at 27 months of age for tumor screening by histologic analysis (*brca2 +/+;tp53 +/+*, n = 6; *brca2 +/m;tp53 +/+*, n = 6; and *brca2 m/m;tp53 +/+*, n = 6). Zebrafish were euthanized and processed for histology as described above.

**Criteria for exclusion from study.** 24 *tp53 +/m* zebrafish and three *tp53 +/+* zebrafish from the study populations were not included in analyses; exclusion of specimens was generally based on gross or histologic evidence of post mortem autolysis. In addition, five tumor-bearing zebrafish were excluded from the study after histologic review. Two zebrafish exhibited extensive autolysis and could not be analyzed for tumor development. Three female zebrafish exhibited sarcoma development in the ovary in association with extensive granulomatous inflammation. Sarcoma development in the ovary can occur in zebrafish as a sequela to chronic granulomatous oophoritis , and this possibility was considered to be a confounding factor that warranted exclusion of these cases from the study.

**Image acquisition and processing.** Histologic slides were evaluated with an Olympus BX43 microscope (Olympus Corporation, Center Valley, PA), and histologic images were captured with a DP26 digital camera (Olympus) and cellSens Entry microscope imaging software, version 1.5 (Olympus). Histologic images were minimally processed with Adobe Photoshop CS3, version 10.0.1 (Adobe Systems Incorporated, San Jose, CA).

**Genotyping**. Live zebrafish were genotyped for *brca2* and *tp53* as previously described . For the majority of animals, fin clip specimens were also collected at the time of death to confirm the *brca2* and *tp53* genotypes. In some instances, genotyping was achieved by sequencing over the mutation sites as described below.

**LCM and DNA extraction from paraffin-embedded zebrafish tissues**. 7-μm unstained tissue sections were prepared (Histoserv, Inc., Gaithersburg, MD) on PEN-membrane glass slides (Fisher Scientific, Pittsburg, PA), and routinely processed for hematoxylin staining. Tumor and normal tissue specimens were individually collected with the LMD 6000 microdissection system (Leica Microsystems, Bannockburn, IL). Multiple tumors from individual zebrafish were collected and analyzed as separate samples. LCM and DNA extraction were not performed on tumors of insufficient size for isolation. DNA specimens from microdissected tissues were extracted with the Ambion® RecoverAll™ Total Nucleic Acid Isolation Kit (Life Technologies Corporation, Grand Island, NY) according to the manufacturer’s protocol. DNA concentrations were determined with the Nanodrop spectrophotometer (ThermoScientific, Wilmington, DE). Purified DNA samples from LCM-collected tissues were subsequently used for LOH and CpG methylation analyses.

**Loss of heterozygosity (LOH) analyses.** LOH analyses were achieved by sequencing over the *brca2^Q658X^* and *tp53^M214K^* mutation sites. Both mutations are single base pair changes that were used to identify the wildtype and mutant alleles in sequence analyses from all specimens. For PCR amplification of DNA samples extracted from paraffin-embedded tissues, primers were designed flanking the *brca2^Q658X^* and *tp53^M214K^* mutation sites. PCR amplicons were purified with the QIAquick PCR purification kit (QIAGEN Inc., Valencia, CA), according to the manufacturer’s protocol, sequenced, and analyzed for the presence of the *brca2* and *tp53* wildtype and mutant alleles. For some tumor specimens, sequencing was additionally performed for single nucleotide polymorphisms (SNPs) located in 5’ and 3’ portions of the gene to confirm LOH status.

**CpG island identification and Pyrosequencing methylation detection assay.** The zebrafish *brca2* promoter has not been characterized, but a CpG island as determined by NCBI algorithm is predicted to occur 306 base pairs upstream of the 5’ position of the translational start codon for *brca2* on chromosome 15 (*Danio rerio* genome version Zv9). The NCBI algorithm for defining CpG islands using relaxed criteria included the following parameters: 200 bp minimum length; at least 50% G + C content; at least 0.60 observed CpG/expected CpG ratio; and merging of islands that are ≤ 100 bp apart. This algorithm was based on the methodology described by Takai and Jones .

All specimens analyzed for CpG methylation were collected from *brca2 +/m;tp53 +/m* zebrafish, and each tumor exhibited LOH for the wildtype *tp53* allele. Purified DNA samples from LCM-collected normal and tumor tissues were analyzed for CpG methylation by Pyrosequencing (Laboratory of Molecular Technology, Science Applications International Corporation, Frederick, MD). PCR reactions were optimized with wildtype zebrafish DNA collected and purified from paraffin-embedded tissues. DNA from normal tissue samples (from either fin clip or LCM-collected normal tissue) and tumor samples were subjected to bisulfite conversion, PCR amplification, and Pyrosequencing analysis. Four assays were performed for each sample.

**Statistical analyses.** To compare the incidence of tumor development in male and female *tp53 +/m* zebrafish, the number of zebrafish that developed one or more tumors, versus the number of zebrafish that did not develop tumors, were grouped by sex and by *brca2* genotype. These proportions were compared in pairs by Fisher’s exact test, and P < 0.05 was accepted to indicate statistical significance (Table 1). Animals for which the sex was not determined were excluded from this analysis. The *brca2 m/m;tp53 +/m* cohort was not analyzed, since no females are present in this group (Table 1).

To compare the incidence of malignant tumors among *tp53 +/m* zebrafish, the number of animals that developed at least one malignant tumor, versus the number of animals that developed only benign tumors or did not develop tumors, were grouped by *brca2* genotype. These proportions were compared in pairs by Fisher’s exact test, and P < 0.05 was accepted to indicate statistical significance (Table 1).

To compare the incidence of MPNST among *tp53 +/m* zebrafish that developed malignant tumors, the number of animals that developed at least one MPNST, versus the number of animals that developed any other malignant tumor type, were grouped by *brca2* genotype. These proportions were compared in pairs by Fisher’s exact test with Mehta’s correction, and P < 0.05 was accepted to indicate statistical significance (Table 3). The same approach was used to compare the incidence of undifferentiated sarcoma among *tp53 +/m* zebrafish (Table 3).

1. Shive HR, West RR, Embree LJ, Azuma M, Sood R, et al. (2010) brca2 in zebrafish ovarian development, spermatogenesis, and tumorigenesis. Proc Natl Acad Sci U S A 107: 19350-19355.

2. Kent M.L. SJM, Matthews J.M., Fournie J.W., Murray K.N., Westerfield M. (2012) Diseases of Zebrafish in Research Facilities. ZIRC Health Services Zebrafish Disease Manual: Zebrafish International Resource Center.

3. Berghmans S, Murphey RD, Wienholds E, Neuberg D, Kutok JL, et al. (2005) tp53 mutant zebrafish develop malignant peripheral nerve sheath tumors. Proc Natl Acad Sci U S A 102: 407-412.

4. Takai D, Jones PA (2002) Comprehensive analysis of CpG islands in human chromosomes 21 and 22. Proc Natl Acad Sci U S A 99: 3740-3745.
